# Case of Hæmorrhage

**Published:** 1839-05-18

**Authors:** J. Strobhart


### CASE OF HÆMORRHAGE.

By J. Strobhart, M.D.

To the Editors of the Medical Examiner.

Gentlemen,—The following case, extracted from my note book, is one so singularly illustrative of the hæmorrhagic constitution, that I have reported it for your Journal; and, if its insertion will not exclude matter of paramount importance, you will please give it a place in your interesting columns.

I am, gentlemen,

Very respectfully, yours, &c.

J. Strobhart.

*Grahamville, (S. C.,) May 10th,* 1839.

The subject of this case, a young woman, about nineteen years of age, of full habit, approaching to *embonpoint,* and apparently of good constitution, was suddenly taken, on the third of March last, with profuse hæmorrhage from the nose, for the relief of which I was called.

Upon my arrival, I found that she had lost about sixteen ounces of blood, and as it still continued to flow, with a full, strong, and slow pulse, I immediately placed her in an elevated posture, and abstracted ℥xvi. from the arm. The epistaxis being now reduced to an almost imperceptible oozing, the nostril was plugged with a dossil of lint, and having directed warm stimulating pediluvia, to be followed by a brisk purgative, I left her doing well.

Six hours subsequently, I was summoned in great haste to arrest the bleeding from the arm, which, after many unsuccessful efforts, was finally effected, and the patient again left without a bad symptom. I, however, began to suspect the peculiar disposition which was subsequently so fully developed: for I am generally very particular in closing the orifice after venesection, and felt satisfied that the recurrence of hæmorrhage could not be imputed to the slovenly performance of this operation.

Five days now elapsed without hearing any thing from my patient. Upon the sixth, however, my services were again required, when I found her in the following situation. Head and face very much swollen; conjunctiva injected; pulse full, slow, but compressible; lower lip very much tumefied, so much so as to be completely everted, and presenting the appearance of so much raw flesh; tongue covered with ecchymosed patches, elevated one-eighth of an inch above the healthy surface. Struck by this singular phenomenon, I extended my examination farther, and discovered that the whole lining membrane of the mouth, gums, velum pendulum palati, and, as far as could be seen, into the fauces, were completely occupied with similar tumefactions, some isolated, and presenting the appearance of that affection of the conjunctiva, termed chemosis; others running, as it were, into each other, and forming one continued ecchymosis; from all of which, the blood was to be seen oozing in such quantities, as, literally speaking, to support a profuse hæmoptysis. No parallel to this case have I ever seen on record, and the only one with which it can be allied, is that cited by Professor Chapman, in his lecture on hæmorrhage, where a similar oozing took place from the gums, (see No. 2, Vol. II., of this Journal.) But here, both gums and teeth presented a healthy appearance upon inspection.

The treatment, on this occasion, was merely tentative; for fearing to resort again to venesection, it consisted chiefly of gentle evacuants, and a gargle composed of a strong decoction of the quercus tincturia.

I was, however, as much delighted as astonished, to find that in a few days every symptom yielded to this treatment. The oozing of blood gradually diminished, and the ecchymosis slowly, but progressively contracted, till every vestige of them was removed, and the patient, apparently, again restored to health. But, to use the language of Hunter, the necessary action had not yet taken place. And here I would remark, generally, upon the impropriety of arresting hæmorrhages of this nature, ere the peculiar disposition, so to speak, has been gratified. For I apprehend that the blood, like the current which meets with obstructions in its onward course, may be thrown back, as it were, upon some more important organ, and serious disorganization be the consequence. Such, indeed, had like to have been the result in this instance, for forty-eight hours had scarcely elapsed, when a copious hæmaturia supervened, which, after resisting many of my best directed efforts, finally yielded to full doses of ipecacuanha, followed by opiates.

So much space has already been occupied with the detail of this case, that my remarks must necessarily be brief, and confined to a few of its most prominent features.

Similar attacks of epistaxis are frequently met with. The recurrence of hæmorrhage, after venesection, is, also, a matter of common occurrence. But the phenomena exhibited by the mouth of this patient, were of the most novel and alarming nature. Never having seen such described, I candidly confess that I was very much at a loss for a proper pathology; and the habit of the patient being such as to induce a suspicion of apoplexy, I was at the first *coup d'œil* disposed to class it with a case which I have heard Professor Jackson relate to his class, which is substantially as follows:—"A young gentleman, upon rising in the morning, had frequently had his attention attracted to a livid spot upon his face; no solution of which could be given, till an unexpected influx of company at the house in which he lodged, rendered it necessary for him to take a bed-fellow, who being aroused in the night by his convulsive struggles, communicated the alarm to the family, when a physician was called, and the enigma solved." Now I can readily conceive, that if a strong determination of blood to the head, be capable of producing such extravasations about the face, the loose and delicate tissues, which enter into the formation of the mouth and its contents, yielding much more readily to the vis a tergo, would admit the blood into their interstices, and form the elevated patches I have attempted to describe. More mature reflection, however, soon determined me to rank it among the other hæmorrhages from mucous surfaces; and though it may differ in a few unimportant particulars, its true analogue is to be found in the case cited by Professor Chapman, as witnessed by Dr. Dewees and himself, and to which allusion has been already made. Indeed, I have but little doubt, that if the stomach of an individual, suffering under an acute attack of hæmatemesis, could be exposed to view, before the blood had retired from the capillaries into the large venous trunks, which always takes place ere an autopsy can be effected, its mucous coat would be found to exhibit very similar phenomena to that of the mouth of my patient.

With regard to the treatment here adopted, I have but little to say, it being remarkable only for its efficiency. I would, however, avail myself of the opportunity to recommend the use of ipecacuanha in all hæmorrhages from vital organs, and more especially those whose sympathies are easily elicited by impressions upon the stomach.

There is yet another circumstance to which I cannot forbear alluding for a moment, *i. e.* to the fact of the blood having been distinctly seen to escape, as it were, from so many separate pores,—an observation strongly corroborative of the anastomotic or exhalent doctrine of hæmorrhage, as taught by Professor Chapman; and I am glad to have it in my power to add another link to the long chain of evidence he has adduced in support of that theory.

---

The Physicians of Ohio are about to hold a Medical Convention at Cleveland, in that State.

